# Myeloid-Derived Suppressor Cells and Pulmonary Hypertension

**DOI:** 10.3390/ijms19082277

**Published:** 2018-08-03

**Authors:** Andrew J. Bryant, Borna Mehrad, Todd M. Brusko, James D. West, Lyle L. Moldawer

**Affiliations:** 1 Division of Pulmonary and Critical Care Medicine, Department of Medicine, University of Florida College of Medicine, Gainesville, FL 32610, USA; borna.mehrad@medicine.ufl.edu; 2 Department of Pathology, Immunology and Laboratory Medicine, University of Florida College of Medicine, Gainesville, FL 32610, USA; tbrusko@ufl.edu; 3 Department of Medicine, Vanderbilt University School of Medicine, Nashville, TN 37232, USA; j.west@vanderbilt.edu; 4 Department of Surgery, University of Florida College of Medicine, Gainesville, FL 32610, USA; lyle.moldawer@surgery.ufl.edu

**Keywords:** pulmonary hypertension (PH), myeloid-derived suppressor cells (MDSC), monocytic-MDSC (Mo-MDSC), polymorphonuclear-MDSC (PMN-MDSC), dendritic cells (DC), macrophages (MΦ), arginase 1 (Arg1), inducible nitric oxide synthase (iNOS), C-X-C motif chemokine receptor type 2 (CXCR2)

## Abstract

Myeloid–derived suppressor cells (MDSCs) comprised a heterogeneous subset of bone marrow–derived myeloid cells, best studied in cancer research, that are increasingly implicated in the pathogenesis of pulmonary vascular remodeling and the development of pulmonary hypertension. Stem cell transplantation represents one extreme interventional strategy for ablating the myeloid compartment but poses a number of translational challenges. There remains an outstanding need for additional therapeutic targets to impact MDSC function, including the potential to alter interactions with innate and adaptive immune subsets, or alternatively, alter trafficking receptors, metabolic pathways, and transcription factor signaling with readily available and safe drugs. In this review, we summarize the current literature on the role of myeloid cells in the development of pulmonary hypertension, first in pulmonary circulation changes associated with myelodysplastic syndromes, and then by examining intrinsic myeloid cell changes that contribute to disease progression in pulmonary hypertension. We then outline several tractable targets and pathways relevant to pulmonary hypertension via MDSC regulation. Identifying these MDSC-regulated effectors is part of an ongoing effort to impact the field of pulmonary hypertension research through identification of myeloid compartment-specific therapeutic applications in the treatment of pulmonary vasculopathies.

## 1. Introduction

Myeloid-derived suppressor cells (MDSCs) were initially described as myeloid cells capable of suppressing T cell proliferation in vitro but are now known as key participants in a number of physiologic and pathophysiologic conditions. Whether granulocytic (most abundantly represented as polymorphonuclear neutrophils) or mononuclear (primarily monocytes), distinct classes of myeloid cells ultimately originate from three simple observations: (1) cell morphology, which is largely undisputed, (2) lineage-tracing, and (3) cell function, a frequent point of contention. MDSCs, and the controversy surrounding this population of leukocytes, are examples of the latter.

Since their original discovery over a decade ago, discussion of MDSCs remains provocative due to their subgroup phenotypic similarities to granulocytes (polymorphonuclear MDSCs; PMN-MDSCs) and monocytes (monocytic MDSCs; Mo-MDSCs). Nonetheless, in states of sustained inflammation—such as chronic infection and cancer—growth factor, cytokine, and chemokine signaling patterns favor the release into circulation of immature myeloid cells that operatively inhibit acquired immune cell responses and enhance cellular proliferation and angiogenesis [1]. This MDSC signature has been described in a number of pulmonary infectious diseases, including: *Pseudomonas* and endemic fungal pneumonia [2,3], tuberculosis [4,5], opportunistic *Pneumocystis jiroveci* pneumonia [6], and influenza [7]. More recently, however, MDSCs have been recognized as playing a critical role in the pathogenesis of other noninfectious lung diseases, such as chronic obstructive pulmonary disease, asthma, and cystic fibrosis [8]. To date, activated MDSCs have been documented in patients with pulmonary hypertension secondary to congenital heart disease, with cell count in peripheral blood strongly correlated with the severity of pulmonary artery pressure elevation [9]. Although a mechanism has yet to be fully developed, we recently demonstrated a potential role for—specifically—PMN-MDSCs in the pathogenesis of pulmonary hypertension related to models of both chronic hypoxia exposure and pulmonary fibrosis [10]. 

Given the immature state of MDSC-related research, a major point of contention remains the discernment of the characteristics setting apart MDSC subpopulations (Mo-MDSCs and PMN-MDSCs) from their morphologically similar innate immune cells (monocytes and neutrophils, respectively). In humans, the distinction is relatively straightforward. Mo-MDSCs and monocytes are distinguished based upon MHC class II expression; Mo-MDSCs have the phenotype CD11b ^+^ CD33 ^+^ CD14 ^+^ CD15 ^−^ and HLA-DR ^−^, whereas monocytes are HLA-DR ^+^ [11]. PMN-MDSCs and neutrophils share a phenotype (CD33 ^+^ CD11b ^+^ CD14 ^−^ CD15 ^+^ CD66b ^+^), however, differences in Percoll density gradients easily distinguish neutrophils (high density) from PMN-MDSCs (low density, with suppressive capability) [12]. Furthermore, transcriptomic analysis has revealed specific signatures identifying neutrophils, PMN-MDSCs, and even tumor-associated neutrophils (TANs) [13].

In mice, Mo-MDSCs are defined as CD11b ^+^ Ly6C^hi^Ly6G ^−^ cells with low granularity, discriminated from monocytes by lack of surface markers CD11c and MHC II, and from macrophages by absence of F4/80 [1]. Specific markers, outside of functional assessment, remain elusive in distinguishing murine PMN-MDSCs from granulocytes, except perhaps related to expression of key metabolic enzymes necessary for facilitating immune escape [14].

The goal of this review is to summarize the literature on the role of MDSCs in the pathogenesis of pulmonary hypertension, focusing on the myriad shared molecular and cell-specific pathways involved in both pulmonary vascular remodeling and MDSC regulation. 

## 2. Pulmonary Hypertension and Myeloid Cell Disorders

In order to establish the role of a specific circulating cell population, such as MDSCs, in the development of pulmonary hypertension, it is useful to first examine broadly the context of myeloid cells in pulmonary vascular disease. To this end, we survey the occurrence of myeloid cell changes in pulmonary hypertension (primarily pulmonary arterial hypertension, PAH), but also examine pulmonary vascular disease in pathologic states of myeloid activation or dysfunction (myelodysplastic syndromes), and—importantly—discuss the effect of stem cell transplantation on disease states associated with lung vessel remodeling. 

### 2.1. Stem Cell Transplantation and Pulmonary Hypertension

Hematopoietic stem cell transplantation (HSCT)—a common treatment for malignant hematologic disease—is frequently considered as a contributor to the development of pulmonary hypertension. Support for a potential causal role in pulmonary artery pressure elevation in this condition, however, is confounded by several factors: chemoradiation injury resulting in occlusive vasculopathy [15], pulmonary hypertension associated with bronchiolitis obliterans [16], and pulmonary thromboembolic disease complicating the use of some immunobiologic agents, such as the tyrosine-kinase inhibitor dasatinib [17].

Although associated with adverse vasculopathic injuries and employed in the treatment of selective disease states that are mainly rheumatologic, there may be beneficial effects of HSCT on the pulmonary circulation. For example, in patients with systemic sclerosis, autologous HSCT was found to be associated with stabilization of pulmonary hypertension in affected patients [18]. Additionally, a 5-year post-transplant follow-up study of this same patient cohort demonstrated a trend towards improved lung function parameters, such as the diffusing capacity of lung for carbon monoxide (DLCO) [19], while a more recent clinical trial showed that, in patients with scleroderma, stem cell transplantation can prevent the development of pulmonary hypertension [20]. Similar disease remission following HSCT has been noted in patients with pulmonary hypertension secondary to systemic lupus erythematosus [21,22]. Finally, in a case report of a patient with treatment-refractory sickle cell anemia, reversal of precapillary pulmonary hypertension was found upon undergoing haploidentical non-myeloablative peripheral blood stem cell transplantation [23]. 

In support of a potentially protective role for bone marrow transplant in the treatment or prevention of pulmonary hypertension, multiple animal models have demonstrated that bone marrow-derived cells ameliorate elevated pulmonary pressures. For example, one study using the monocrotaline model of pulmonary hypertension examined the role of endothelial progenitor cell adoptive transfer in the prevention of disease, finding that early administration of cells completely halted the development of pulmonary hypertension, when compared to infusion of normal fibroblasts [24]. Subsequent follow-up research on the use of these endothelial-like cells in small animal models has shown that recruitment and metabolic factors—such as erythropoietin receptor and nitric oxide synthase, respectively—are necessary for the appropriate integration of these cells into the distal arterioles [25,26]. These multiple contributory factors may in part explain the disparate findings amongst groups of whether bone marrow-derived cells contribute solely to hypoxia-induced pulmonary hypertension, as opposed to mechanical or chemical-related pulmonary vascular injury and repair [27,28]. Regardless, whole bone marrow transplant, after total body irradiation, has been demonstrated to be protective against the pulmonary hypertension phenotype in multiple species, including mice [29], rats [30], and dogs [31]. The most definitive evidence supporting the role of bone marrow-derived cells in the development of pulmonary hypertension and pulmonary vascular remodeling comes from recent studies, in which bone marrow chimeric mice were generated from animals with canonical mutations predisposing them to human PAH, including bone morphogenetic protein receptor type II (BMPR2) [32] and calveolin–1 (Cav1) [33]. Collectively, these data are suggestive of a tissue-specific influence by the myeloid compartment in the initiation and maintenance of some forms of elevated pulmonary pressures. 

### 2.2. Myelodysplastic Syndromes and Pulmonary Hypertension

There is a universally poor prognosis associated with the development of elevated pulmonary pressures in the setting of myeloproliferative disorders, a complication affecting roughly 40% of such patients, as noted in a retrospective chart review [34,35]. Among the reported causes of pulmonary vascular disease, patients exhibit hematopoietic infiltration of pulmonary parenchyma and hypercoagulability, leading to chronic thromboembolic pulmonary hypertension and thrombocytosis [36]. Intriguingly, myelofibrosis appears to be unique among chronic myeloproliferative disorders in its consistent association with elevated pulmonary artery pressure. In one study, compared to patients with chronic myelogenous leukemia and aplastic anemia, those with myelofibrosis alone had significantly greater right-ventricular pathology by echocardiogram assessment [37]. It remains unclear, however, if this association represents a causal relationship, or merely a secondary link between pulmonary hypertension and primary myelofibrosis. The latter conclusion is supported by the lack of reported association between development of pulmonary hypertension and the allelic burden of common Janus kinase 2 (JAK2) allelic variant (V617F), which is responsible for many myeloproliferative neoplasms [38,39].

Analogous to the above-described examples, allogenic stem cell transplant was shown to attenuate pulmonary hypertension associated with myelofibrosis [40] in a patient with prohibitive comorbidities and drug–drug interactions precluding use of traditional vasodilator therapies. This case is consistent with what is known regarding chronic myeloproliferative disorders associated with pulmonary hypertension, whereby there are multiple attributable causes described [41], similar to the noted heterogeneous etiologies of pulmonary hypertension after treatment with bone marrow transplant [42,43]. 

### 2.3. Abnormalities in Myeloid Cells in Pulmonary Hypertension

There is ample evidence describing pulmonary vascular disease, PAH in particular, as a systemic disorder. In particular, PAH is associated with intrinsic alterations in the myeloid compartment. Patients with PAH show evidence of decreased monocyte activation through the inhibition of nuclear factor kappa B cell (NF-ĸB) signaling, interleukin-6 (IL-6), CC chemokine ligand-3 (CCL3, previously macrophage inflammatory protein 1-alpha), and vascular endothelial growth factor (VEGF) [44]. In addition, they display an enhanced neutrophil inflammatory response to various stimuli that is tempered by in vitro incubation with prostacyclin analogue iloprost [45]. Furthermore, bone marrow-derived proangiogenic progenitor (CD34 ^+^ CD133 ^+^) cells are elevated in the circulation of PAH patients [46]; interestingly, the subclinical myeloproliferative process is also present in nonaffected family members of PAH patients, and it is associated with an increase in endothelial cell production of hypoxia-inducible factor response element chemokines, such as stromal cell-derived factor 1 (SDF1) [47]. 

In a landmark study, Asosingh and Erzurum’s group elaborated upon the cellular mechanism of pulmonary vascular disease attributed to intrinsic myeloid cell abnormalities [48]. As reported, they first isolated CD133 ^+^ bone marrow progenitor cells from patients with PAH and healthy controls, injecting the populations into NOD-SCID mice. They then assessed for differences in right ventricular hypertrophy, pulmonary vascular remodeling, and mortality between groups, finding that only in mice transplanted with cells from patients with PAH did the disease develop. These findings are consistent with others reporting a supporting role of circulating monocytic lineage precursors—alternatively referred to as fibrocytes—in hypoxia–induced large animal models of pulmonary hypertension [49]. 

Perivascular cellular recruitment—especially of macrophages—has been described in PAH [50], with the process facilitated through a diverse array of mediators, including leukotriene signaling [51], and granulocyte-macrophage colony-stimulating factor (GM-CSF) production [52], in patients that carry a common mutation in BMPR2. Subsequently, the local inflammatory milieu directs a shift from an M1 (inflammatory) to an aberrant M2 (reparative) phenotype, promoting deleterious vascular healing [53]. This is similar across other forms of pulmonary hypertension, where type 2 inflammation is integral to the proliferative vascular response, such as in schistosomiasis-mediated vascular disease [54]. Of relevance to this point, bone marrow-derived mononuclear cells, administered 4 weeks after monocrotaline administration, improved pulmonary hypertension through both increased VEGF expression [55] and inhibition of soluble inflammatory mediators [56]. In contrast, growth factors associated with monocytic expansion, such as GM-CSF, increase inflammatory cell recruitment to the lungs in mice with the BMPR2 mutation, exacerbating pulmonary hypertension [52]. This effect may be attributable to macrophage activation in a paracrine-dependent manner, which mediates local pulmonary vessel smooth muscle cell changes directly [57].

Due to this highlighted function in cellular trafficking and accumulation that is critical to advancement of pulmonary vasculopathies, chemokine receptors play a major role in progression of pulmonary hypertension. C–C chemokine receptor 2 (CCR2), the receptor for CC chemokine ligand 2 (CCL2), is a cell surface signaling protein which facilitates the egress of mononuclear cells from the bone marrow into the circulation, as well as the rolling, adhesion, and diapedesis of circulating cells into the lung vascular bed. CCR2-deficient mice have an expected decrease in recruitment of inflammatory monocytes to the lung during chronic hypoxic stress, associated with worsening of pulmonary hypertension [58]. 

Polymorphonuclear neutrophils—especially direct neutrophil interactions with lymphocytes—have been shown to be important in the development of pulmonary hypertension. An elevated neutrophil-to-lymphocyte ratio in the peripheral blood is associated with a poor prognosis in pulmonary hypertension patients [59], speculated to be due, in part, to neutrophil extracellular trap promotion of inflammatory angiogenesis [60]. These findings are consistent with established experimental data derived from sheep and rabbit models of disease, which demonstrate that granulocyte depletion is protective against pulmonary hypertension [61,62]. A similar effect on lung disease is seen in mice with antagonism of a chemokine receptor, C–X–C chemokine receptor 2 (CXCR2), expressed predominantly on circulating granulocytes and endothelial progenitor cells. For example, in BMPR2 mutant mice—with deletion of BMPR2 in only the vascular endothelium—treatment resulted in protection against pulmonary hypertension progression [63,64]. This protection was strongly associated with a decrease in lung myeloperoxidase and vascular barrier permeability. Interestingly, neutrophil populations were relatively unaffected by CXCR2 inhibition, suggesting another cell type of interest contributing to vascular disease, such as PMN-MDSC. Yet, a more recent report has described a decrease in neutrophils in PAH patients’ lung tissue samples associated with a concomitant increase in T cell subsets, including γδ cells, and plasmacytoid dendritic cells [65], providing a potential innate-to-adaptive immune system link in disease progression. 

Functionally, local differentiation or maturation of myeloid cells influences the development of pulmonary hypertension to a larger degree than simple trafficking to the pulmonary vasculature of various bone marrow-derived cell populations. Although myeloid cells have been shown to worsen the development of pulmonary vascular remodeling in response to chronic hypoxia and monocrotaline injection, inhibiting downstream maturation of myeloid cells within tissue can protect against this outcome [66]. While this phenomenon is thought to be mitigated in large part by the cellular cross-talk between immune and endothelial cells, maturation of myeloid cells is also likely to influence signaling with alternative cell types, including T lymphocytes, in the lung perivascular space. Such accumulation and activation of the adaptive immune system represents a potential feed-forward mechanism in the lungs of diseased animals, and potentially humans; increased myeloid cell trafficking leads to low-grade adaptive immune cell inflammatory signaling, attendant peripheral cell recruitment, and worsening of disease [67]. This may in part explain how soluble factors, such as IL-6, contribute to T helper 17 cell (T_H_17) activation and the M2-like macrophage response in hypoxia-induced pulmonary hypertension [53,68], as discussed below.

## 3. Myeloid-Derived Suppressor Cells and Pulmonary Hypertension

**Figure 1:**
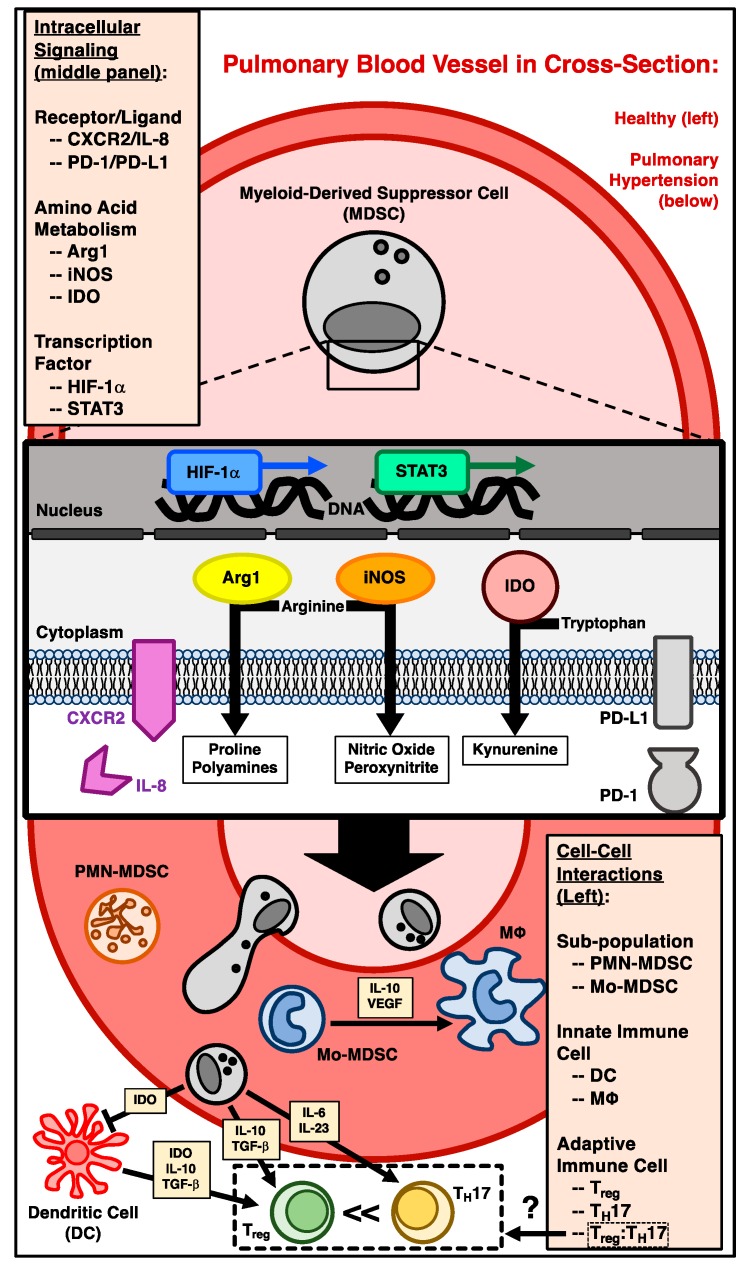
Illustration detailing potential myeloid-derived suppressor cell (MDSC) contributions to pulmonary vascular remodeling and development of pulmonary hypertension (PH). Displayed are proposed intracellular and intercellular MDSC-mediated mechanisms in the development of PH. These include, although are not limited to: receptor/ligand interactions (CXCR2/IL-8 and PD-1/PD-L1), regulation of amino acid metabolism favoring cellular proliferation and aberrant wound repair (arginine via Arg1 and iNOS, and tryptophan through IDO), and transcription factor stabilization and activation (HIF-1α and STAT3). Cell–cell interactions include molecular cross-talk between: subpopulations of MDSC (PMN-MDSC and Mo-MDSC), innate immune cells (MΦ and DC), and—finally—adaptive immune effector cells (Treg and T_H_17). The disarrangement of the latter cell groups’ ratio (Treg:T_H_17) is likely of particular importance in influencing the microenvironment favoring pulmonary vascular remodeling and PH. Abbreviations: CXCR2 (C-X-C motif chemokine receptor type 2); IL-8 (interleukin 8); PD-1 (programmed cell death protein-1); PD-L1 (programmed death-ligand 1); Arg1 (arginase-1); iNOS (inducible nitric oxide synthase); IDO (indoleamine-pyrrole 2,3-dioxygenase); HIF-1α (hypoxia-inducible factor-1 alpha); STAT3 (signal transducer and activator of transcription 3); PMN-MDSC (polymorphonuclear MDSC); Mo-MDSC (monocytic MDSC); DC (dendritic cell); MΦ (macrophage, particularly M_2_-polarized); Treg (regulatory T cell); T_H_17 (T helper 17 cell); IL-10 (interleukin 10); VEGF (vascular endothelial growth factor); IL-6 (interleukin 6); IL-23 (interleukin 23); TGF-β (transforming growth factor beta).

Numerous myeloid cells likely contribute to the development of pulmonary hypertension. Though there is an acknowledged role for a diverse number of cell types in disease pathogenesis, including eosinophils [69], fibrocytes [70], dendritic cells [65], endothelial progenitor cells (both early [71] and late [72,73]), and mast cells [74], the remainder of this analysis focuses exclusively on the role of MDSCs in pulmonary hypertension. Specifically, attention is drawn to shared intracellular (molecular) and intercellular (cell–cell) mechanistic pathways between pulmonary hypertension and MDSC-mediated disease development (refer to Figure 1 and Table 1 for a detailed summary). Finally, the MDSC contribution to metabolic changes within the tissue microenvironment, including effects on vascular endothelial and smooth muscle cells, is also considered.

### 3.1. Molecular Mechanisms

#### 3.1.1. C–X–C Motif Chemokine Receptor Type 2 (CXCR2)

As described earlier, the studies by Burton and Budd [63,64] define a significant role for CXCR2–mediated accumulation of circulating leukocytes in pulmonary hypertension. Initially exploring the link between accrual of leukocytes within the lung and subsequent vascular injury repair in BMPR2 heterozygous null mouse models, they demonstrated that loss of BMPR2 expression within the vascular endothelium leads to increased susceptibility to pulmonary inflammatory stimulus through worsening vascular barrier permeability [63]. They then went on to show that, with administration of a CXCR1/2 inhibitor, not only was accumulation of leukocytes attenuated, but the pulmonary hypertensive response was corrected as well [64]. It remains to be elucidated, however, which tissue-specific expression of CXCR2—myeloid, endothelial, or epithelial cell—is most contributory to disease progression. Of relevance to this point, at least one additional study has demonstrated a protective role for CXCR2 overexpression in endothelial cells in the monocrotaline rat model, with marked decrease in neutrophil accumulation to the lung and decreases in interleukin-8 (IL-8) expression within the lung [75]. 

As mentioned, we have demonstrated an association between decreased MDSC homing to the inflamed lung and attenuation of pulmonary vascular remodeling with administration of a selective CXCR2 inhibitor. There are additional supportive data, generated primarily from the cancer and autoimmunity literature, that establishes a role for CXCR2-mediated trafficking and activation in PMN-MDSC-mediated pathology. For example, in an inflammatory colitis and colon cancer model, CXCR2 null mice were protected against development of disease, with tumor progression restored only after adoptive transfer of activated MDSCs [76]. In another model examining pancreatic cancer, it appeared that targeting CXCR2 expression by immune cells was protective against malignant progression and metastasis, primarily through promotion of effector T lymphocyte—CD8 ^+^ T cell—activity [77]. The same phenomenon has been observed in bladder cancer [78]. 

Although CXCR2 is expressed highly by circulating neutrophils in relevant models of comparison, such as cancer, it is primarily and functionally associated with upregulation on PMN-MDSCs [12]. Additionally, other cell-surface chemokine receptors commonly expressed by Mo-MDSC, such as CCR2 and CX3CR1, are highly expressed in circulating immune cells accumulating within the lungs of mice with chronic hypoxia-induced pulmonary hypertension [79]. A direct role, however, for secreted CCR2 and CX3CR1 ligands in regulating the hemodynamic changes, beyond recruitment of inflammatory monocytes and indirect influence on pulmonary artery smooth muscle cell hyperproliferation, remains unproven [80]. 

#### 3.1.2. Arginase–1 (Arg1)

In the original report by Yeager and colleagues, MDSCs detected in the peripheral circulation of patients with pulmonary hypertension were noted to have increased Arg1 transcripts, consistent with a detected increase in urea activity from isolated patient samples [9]. Relevant to multiple contributory pathways involved in pulmonary hypertension, arginase is a required initial step in the polyamine production, ornithine flux, and proline synthesis necessary for cellular proliferation and collagen formation. Importantly, arginase activity is known to be elevated in PAH patients with an increase in both arginine clearance and ornithine flux, without alterations in citrulline flux, *de novo* arginine synthesis, or nitric oxide synthesis [81]. Furthermore, experimental hypoxia is known to upregulate arginase activity via hypoxia-inducible factor*–*2 alpha (HIF–2α), with decreased pathologic signs of hypoxia-induced pulmonary vascular remodeling noted upon deletion of Arg1 in pulmonary vascular endothelial cells [82]. These data are consistent with the recent detection of a protective SNP variant in Arg1 that results in decreased arginase activity against development of pulmonary hypertension in at-risk infants with bronchopulmonary dysplasia [83]. The potential therapeutic application of this finding has been demonstrated in rats exposed to monocrotaline, where pharmacologic arginase was inhibited by administration of the small molecular inhibitor Nω-hydroxy-nor-l-arginine (nor-NOHA), ameliorating pulmonary hypertension and diminishing lung tissue remodeling [84]. 

Although recent evidence suggests that Arg1 is neither inherently expressed in MDSCs nor required for MDSC-mediated inhibition [85], Arg1 expression by MDSCs is widely considered the essential feature by which these cells mediate their immunosuppressive role, by mediating arginine depletion and downstream T cell receptor downregulation [86]. This is thought to occur mainly in PMN-MDSCs in common cancer models [87,88]. Thus, it is intriguing to hypothesize that PMN-MDSC arginase activity may contribute to pulmonary vascular remodeling through the cooperative interaction of two distinct mechanisms: pro-collagen production and perivascular fibrosis, and by downstream effects on T lymphocyte cross-talk. 

#### 3.1.3. Inducible Nitric Oxide Synthase (iNOS)

With respect to arginine metabolism, one must consider not only arginase activity, but also substrate use by the various members of the nitric oxide synthase (NOS) family. While endothelial NOS (eNOS) and the vasodilatory properties of nitric oxide are well known to influence pulmonary hypertension [89], only recently has it been demonstrated that myelocytic NOS expression may be necessary in order to prevent pulmonary hypertension in a murine chronic-hypoxia model [90]. In this study, chimeric mice generated from transplanting NOS-deficient bone marrow into wild-type recipients experienced worsened pulmonary hypertension compared to mice transplanted with wild-type bone marrow. Additionally, in the analysis of nitric oxide levels in the bronchoalveolar lavage fluid of patients with idiopathic pulmonary fibrosis, the group found an inverse correlation between levels of the metabolite and pulmonary artery systolic pressures in patients. Adding to the credibility of the findings, these data are consistent with a previous report examining pulmonary hypertension secondary to emphysema [91]. In the latter study, inhibition of iNOS specifically—with an attendant decrease in tissue peroxynitrite concentration—was found to protect against the development of pulmonary hypertension secondary to chronic tobacco smoke exposure. Furthermore, in mice transplanted with bone marrow cells lacking iNOS, pulmonary hypertension developed as expected compared to the control group, proving the myeloid compartment as necessary for disease progression.

Related to these reports, peroxynitrite and free radical maintenance by iNOS is one of the primary mechanisms that MDSCs are shown to directly inhibit T cell function in a localized inflammatory response, such as tumor growth [92]. In a melanoma model, this is known be a VEGF-dependent process. Increased levels of VEGF enabled immune suppression by increasing signal transducer and activator of transcription 3 (STAT3) activation, and reactive oxidative species production, in recruited MDSCs. This established a positive feedback loop of MDSC recruitment and activation. The feed-forward mechanism was interrupted by iNOS inhibitor l-N^6^-(1-iminoethyl) lysine dihydrochloride (l-NIL), which normalized VEGF levels and negated the immunosuppressive capabilities of MDSCs [93]. A similar phenomenon is thought to influence the pathogenesis of pulmonary hypertension [94]. 

#### 3.1.4. Indoleamine-Pyrrole 2,3-Dioxygenase (IDO) 

IDO-mediated tryptophan metabolism is closely related to arginine regulation and regulates the immunosuppressive capabilities of myeloid cells [95]. In addition, endothelial IDO ameliorates experimentally induced pulmonary hypertension via paracrine proapoptotic signaling with pulmonary artery smooth muscle cells [96]. In patients with PAH, however, metabolic profiling has identified tryptophan metabolites to be associated with right ventricular and pulmonary vascular dysfunction [97]. In particular, serum kynurenine—a primary IDO–tryptophan metabolite—is significantly elevated in PAH patients. This suggests potential IDO-metabolite resistance in these patients, given that kynurenine opposes pulmonary artery vasoconstriction via nitric oxide-mediated vasodilation, acutely decreasing mean pulmonary artery pressure [98].

Indirectly related, transcription factor-dependent IDO expression mediates the immunosuppressive effect of MDSCs in a breast cancer model, with IDO blockade leading to the inhibition of effector T cell response and improvement in disease-related outcomes [99,100]. Unlike previously discussed mechanisms, however, IDO-associated immune escape is predominantly thought to be related to Mo-MDSC pathology (CD14 ^+^ HLA-DR ^−^ peripheral cells in patients with cancer) [101], although this characterization remains a point of debate. Related to the evolving nomenclature of the immune cell population in patients with PAH, a group has recently characterized a novel “fibrocytic MDSC” [102]. These primarily regulate Treg cells through direct contact-mediated IDO upregulation. Other investigators have similarly broadened the role for tryptophan metabolism in MDSC-related immune escape, arguing that it is tumor-expressed IDO that is the primary mechanism regulating immunosuppression within the tumor/tissue microenvironment [103]. It remains to be determined if production of IDO and IDO–tryptophan metabolites by MDSCs or pulmonary vasculature meaningfully contributes to the development of pulmonary hypertension. 

#### 3.1.5. Signal Transducer and Activator of Transcription 3 (STAT3)

Prosurvival proliferative transcription factor activation is implicated in several generic pathologic processes. The STAT family, and STAT3 in particular, are broadly implicated in the pathogenesis of pulmonary hypertension in animal as well as human tissue models. To date, at least one group has firmly demonstrated that inhibition of STAT3-related signaling molecule Pim1 reverses pulmonary vascular remodeling in the rat monocrotaline model [104]. The same investigators also showed that hormone–responsive pulmonary hypertension requires a functional STAT3 signaling axis [105]. Related to this is MDSC regulation through STAT, which has been found to be important in other systemic inflammatory models of disease, such as septic shock, where PMN-MDSC-associated autophagy is regulated directly by STAT3 phosphorylation [106], leading to worsened outcomes. These findings highlight the shared pathways between pulmonary vasculopathies and cancer pathobiology, and they open the door to potential pharmacologic agents available to target STAT-mediated pathways [107]. Many prospective drugs affecting STAT signaling have already undergone intense study as potential therapies targeting MDSC-mediated immune suppression [108], directly applicable to the field of pulmonary hypertension research. 

#### 3.1.6. Hypoxia-Inducible Factor (HIF)

Similar to the regulation of JAK–STAT, acute and chronic changes in the lung microenvironment (such as pH, temperature, and oxygen content) can impact another evolutionarily ancient transcription factor, hypoxia-inducible factor (HIF). This highly conserved protein is stabilized in response to predominantly hypoxic and/or metabolic stress, with both of its main isoforms being implicated in the development of chronic hypoxia-induced pulmonary hypertension: HIF-1α [109] and HIF-2α [110]. Although a thorough review of tissue-specific HIF regulation in lung vascular disease is beyond the scope of the current work, it is relevant to the discussion at hand to mention a major recent discovery in myeloid cell HIF expression’s contribution to pulmonary hypertension. In an elegant series of experiments, Sheikh and colleagues demonstrated that, in a cell autonomous manner, myeloid cells transdifferentiate into, or fuse with, distal pulmonary arteriole smooth muscle cells during hypoxic exposure; it follows that with deletion of a key hypoxia-response element gene in myeloid cells, the pathologic vessel changes are attenuated [111]. This is important, as HIF-1α has been shown to be a primary driver of MDSC differentiation and function, with a shift toward a tumor-associated macrophage phenotype (discussed in the *Cellular Mechanisms* section, below) phenotypic differentiation and activation [112,113,114].

Mediation of such cell–cell interactions by HIF is not limited to a relationship between MDSC and other innate immune cell populations. HIF similarly regulates programmed cell death protein-1 (PD-1)/programmed death-ligand 1 (PD-L1) signaling that functions principally in MDSC and CD8 ^+^ effector T cell direct interactions in patients with obstructive sleep apnea, a common cause of secondary pulmonary hypertension [115]. Such immune checkpoint inhibitors, including PD-L1, are often hypoxia-response element genes themselves. Thus, experimentally blocking these targets with available immunobiologic agents in chronic hypoxia models leads to increased T cell activation, chiefly due to decreased MDSC activity and function [116]. Therefore, the application of these techniques to pulmonary hypertension research is potentially beneficial.

### 3.2. Cellular Mechanisms

#### 3.2.1. Dendritic Cells (DCs)

In patients with PAH, immature DCs—as well as activated CD209 ^+^ DCs—accumulate in the remodeled pulmonary perivascular space [117,118]. The latter study described an increase in activated classical myeloid-derived DCs and nonclassical plasmacytoid DCs derived from the lungs of PAH patients. Accordingly, it remains the predominant thought that DCs and monocytes are actively recruited to the pulmonary vascular microenvironment in the chronic hypoxia model of pulmonary hypertension [119]. This finding is most consistent with the fact that PAH patients also display a decrease in circulating myeloid DCs and monocyte-derived DCs, compared to healthy control subjects [120].

Tolerogenic DC-like cells, which suppress T cell function, have been reported to differentiate from Mo-MDSCs in a mouse model of interstitial lung disease [121]. In cancer models, Mo-MDSCs are thought to undergo a transition to antigen-presenting DC-like cells as part of the increased response to tumor neoantigen and subversion of immune escape [122]. Less in known about direct MDSC-to-DC cross-talk [123], but a well-described consequence of increased MDSC density is an inverse effect on maturity of physically adjacent DCs, an intriguing potential mechanism—and therapeutic application—relating to the presence of immature DCs in patients with PAH [124]. MDSCs reproducibly influence DCs in manner that decreases antigen uptake—with subsequent decreased T cell activation—and skews to an anti-inflammatory cytokine milieu [125,126]. Ultimately, this may prove to be the most important influence on the vascular remodeling potential of MDSCs, as decreased DC production of IL-23 leads to lack of T_H_17 induction [127], skewing the ratio of Treg:T_H_17 in a potentially deleterious direction.

#### 3.2.2. Macrophages

Macrophages are unique in that they are considered the front line of innate immune cells acting in specialized roles, on a spectrum of activities, in response to a panoply of disorders [128]. Macrophages predominantly exist as either derived from recruited “inflammatory” monocytes that undergo transition to macrophages, or as tissue “resident” macrophages that remain in a relatively quiescent state, sensing the immediate environment until provoked by an inflammatory stimulus [129]. As is the case with DCs, macrophages have long been implicated in the immunohistologic pathogenesis of pulmonary hypertension, with large amounts of perivascular macrophages noted in lung samples from patients with PAH, compared to healthy controls [130]. Experimentally, early monocyte/macrophage recruitment is required for hypoxia-induced pulmonary vascular remodeling [131]. BMPR2 mutations contribute to this aberrant trafficking in either an endothelial-specific manner [52] or independently through macrophage BMP pathway dysfunction [57]. Subsequent influence on the polarization of macrophages in the lung remains important, as a shift to an IL-10 productive phenotype has been shown to be protective against hypoxia-induced pulmonary hypertension [80]. This phenotypic plasticity is the primary focus of much of the macrophage-related research in pulmonary circulation research. Currently, the field has moved beyond the earlier described characterization of macrophages as either “inflammatory” (M1) or “repair” (M2), to a distinct profibrotic/proinflammatory amalgam [132]. 

A similar shift from the M1/M2 dichotomy is a well-defined response to MDSC tumor infiltration, first described in oncologic studies, therefore carrying the moniker “tumor-associated macrophage” (TAM) [133]. In isolation, experimental data craft a convincing argument that the relationship between MDSCs and TAMs or TAM-like cells may be potentially beneficial in application to pulmonary hypertension: the MDSC-to-TAM cross-talk facilitates an increase in IL-10 production, a subsequent decrease in IL-12 secretion, and an overall activation of FoxP3 ^+^ Treg populations [134]. Taken together, however, the cumulative effect of *direct* MDSC immune suppression strategies (Arg1, iNOS, and IDO) could simply overpower even the most robust *indirect* macrophage-mediated protective response to ongoing lung tissue injury. Such an effect may favor progression of pulmonary vascular disease. Alternatively, IL-10 may downregulate MHC class II presentation to the cell surface, leading to specific unresponsiveness of T cells to potential alloantigens involved in the development of pulmonary hypertension [135]. More studies are required to make definitive conclusions regarding this topic.

#### 3.2.3. Regulatory T Cells (Treg) and T helper 17 Cells (T_H_17)

Regulation of T lymphocyte populations in patients with pulmonary hypertension is an intriguing area of research in light of known alterations in circulating T cell subsets. An increase in circulating CD8 ^+^ effector T cells and elevation in CD4 ^+^ FOXP3 ^+^ (Treg) cells in patients with PAH [136] have been corroborated in genetic models of pulmonary hypertension [137]. As subsequent studies have shown, while absolute number of individual T-cells may be increased, there is evidence that this may be due in part to a global decrease in T regulatory cell function in those with PAH [138]. This finding illustrates some of the difficulty in interpretation and translation of these complicated findings to humans with disease, as seemingly conflicting reports have described an elevation in *functional* Treg in those with PAH [139]. Nonetheless, it is clear that complete deficiency in T cells predisposes individuals to pulmonary hypertension, as evidenced in athymic nude rats, which develop pulmonary hypertension solely in response to the VEGF receptor block (SU5416) in the absence of usual co-stimuli hypoxia [140]. The picture becomes even more complicated when, in examining in some models of disease, depletion of CD4 ^+^ T_H_2 cells alone ameliorates pulmonary arterial muscularization [141], while in other studies, CD4 ^+^ cell adoptive transfer causes worsening pulmonary hypertension in response to ongoing endothelial injury [142]. 

Potentially reconciling these disparate findings, more recent data have shown that immune reconstitution of T cell-deficient rats with functional Treg prevents pulmonary hypertension [143]. There is also evidence, however, to suggest that this may reflect the Treg:T_H_17 cell balance. Influencing this ratio directly, patients with PAH are noted to have an increase in circulating IL-17 compared to controls [144], perhaps related to tryptophan metabolism [145], although this may simply be present in a subset of patients [146]. Still, these data assume that Treg in patients with PAH are functionally equivalent to those from controls; there is convincing evidence to suggest that there is not only Treg dysfunction in pulmonary hypertension [147], but amplified T_H_17 activation [68]. In reference to the above discussion on the role of macrophages and DCs in pulmonary hypertensive changes, the resulting effects on T cells may also influence accumulation of innate immune cell populations in the perivascular space, leading to increased activation of mediators that can either worsen or improve pathologic pulmonary artery remodeling [67], depending on context.

MDSCs, by definition, regulate T cell proliferation and are known to cause an increase in Treg at the expense of T_H_17 cells [148]. In autoimmunity, though, the opposite has been described: MDSCs, primarily PMN-MDSCs, are associated with an absolute decrease in Treg and an increase in T_H_17 activity, an imbalance that is restored upon MDSC depletion [149] through primarily Arg1-dependent mechanisms [150]. Treg can also recruit and activate MDSCs, although if the function of either is impaired, an increased number of both cell types may accumulate in the tissue and in circulation [103], contributing to disease. Finally, there are a number of ways that identified MDSC and T cell populations may contribute to pulmonary hypertension through many of the previously discussed metabolic or signaling pathways, including Arg1, iNOS, STAT3, and PD-L1/2 activation [151]. Future research will require in-depth study of each of these mediators in relation to adaptive immune system changes in the development of pulmonary hypertension. 

### 3.3. MDSCs and Metabolism

Many of the thus far described molecular pathways and recruited—or transformed—immune cells point to a common MDSC influence on metabolism, with resulting phenotypic changes to the tissue microenvironment in either tumor stroma (in the case of malignancy) or, potentially, endothelial and smooth muscle cells (related to pulmonary hypertension). MDSCs are known to accelerate cancerous growth and, in particular, increase associated epithelial-to-mesenchymal transition through several soluble secreted factors [152]. Within the tumor microenvironment, these changes are fostered by increased oxidative phosphorylation and a shift to aerobic glycolysis as the primary means of energy production (the Warburg effect). This glycolytic shift further drives the increase in immunosuppressive capabilities of MDSCs [153]. Tumor-infiltrating MDSCs also increase fatty acid uptake and oxidation, leading to an increase in the oxygen consumption rate, influenced by local hypoxia and lactic acid accumulation [154]. The process is coordinated in large part by HIF stabilization and Arg1 expression, as previously discussed. 

Through HIF signaling, silent mating type information regulation 2 homolog 1 (sirtuin 1, or SIRT1)—a critical sensor of energy homeostasis—is a primary driver of MDSC differentiation, with deficiency leading to a M2 polarized state. The resulting TAM phenotype is associated with decreased glycolytic activity [155]. Importantly, SIRT1 can serve as a master translator during propagation of acute and chronic inflammatory responses [156]. In support of this concept, mice without myeloid sirtuin 1 display an M1 inflammatory phenotype and delayed progression of tumor growth [155]. Such cross-talk may contribute to transcriptional level control of vascular endothelial proliferation and angiogenesis by niche MDSCs [157], which have previously been shown to be necessary for development of PH [158]. Similar cell–cell communication may also explain why myofibroblasts are known to promote differentiation of MDSC—through signaling proteins S100A8/A9, IL-6, and IL-8—into TAM-like cells [159]. Interestingly, MDSC-derived fibrocytes are promoted by transcription factor Krüppel-like factor 4 (KLF4) during tumor metastasis, boosting tumor growth as they adopt the cell fate [160]. Comparable smooth muscle cell progenitor cells prime muscularization of pulmonary arteries in hypoxic pulmonary hypertension [161], while endothelial cell-derived KLF4 can modulate hyperproliferative vessel changes in pulmonary vascular remodeling [162].

Metabolic changes in the pulmonary arterial tissue bed, related to the development of pulmonary hypertension, have been extensively summarized previously [163,164]. Recently, however, detailed layered transcriptomic and metabolomic analysis of human pulmonary microvascular endothelial cells expressing BMPR2 mutations have described a novel decrease in energy utilization through the Krebs cycle in affected tissue, similar to pathophysiology described in the cancer literature [165,166]. Comparable changes have also been described in smooth muscle cells [167]. Therapeutic applications related to these findings remain in the nascent stage, as much more research is required in the field. For example, although SIRT1 expression in immune cells can lessen unregulated cellular growth, resveratrol—a sirtuin 1 agonist—decreases pulmonary hypertension in the rat monocrotaline model [168], and others [169], in a pulmonary artery smooth muscle cell-specific manner [168].

## 4. Strategies for Therapeutic Targeting of MDSCs in Pulmonary Hypertension

Due to the important role MDSCs play in tumor-induced immunosuppression, these cells could be a promising target for therapy in pulmonary hypertension (refer to summary Table 2). Perhaps most relevant to this discourse is the application of a drug class already in widespread use for patients with pulmonary hypertension—phosphodiesterase-5 inhibitors. Used primarily as a vasodilatory agent, sildenafil is also known to decrease MDSC Arg1 and iNOS expression, leading to decreased immunosuppressive capabilities; increased CD8 ^+^ T cell activation; and reduced tumor metastasis in several cancer models [170,171,172]. Likewise, Tadalafil improves tumor-specific and nonspecific inflammatory responses through decreased immunosuppressive action in patients with either head and neck squamous cell carcinoma or multiple myeloma [173,174]. Since this drug class is already a staple of treatment for pulmonary hypertension, future research should look specifically at combining phosphodiesterase-5 inhibitors with alternative MDSC targets, a portion of which are discussed below.

Blockade of retinoic signal transduction by all-trans retinoic acid (ATRA) induces differentiation of MDSCs to either macrophages or DCs [175], leading to a reduction of MDSC frequencies and improved survival in patients with cancer [176,177]. Importantly, ATRA has been shown to decrease collagen deposition in the rat monocrotaline model of pulmonary hypertension [178]. Decreased muscularization of resistant pulmonary arteries has also been demonstrated with ATRA administration in this model [179], although not always associated with a decrease in the pulmonary hypertension phenotype [180]. 

Since STAT3 is a primary regulator of MDSC-mediated immune escape, inhibition of this transcription factor is an attractive target for the treatment of pulmonary vascular disease. As a logical consequence of this rationale, myeloid-specific targeting of STAT3, through decoy oligonucleotide administration, has already been shown to be successful in the treatment of acute myeloid leukemia in a preclinical model of disease [181]. Additionally, AZD9150, a next-generation antisense oligonucleotide inhibitor of STAT3, has already been tested in a phase I clinical trial of patients with lymphoma and lung cancer [182]. These therapies may prove to be especially helpful in combination with other drugs that target the STAT3 pathway shown to have efficacy in pulmonary hypertension management, such as dehydroepiandrosterone (DHEA) [105]. 

Notably, studies on PD-L1 in pulmonary hypertension development have previously focused on the role of effector T cells and endothelial cells [183], whereas little is known about the role of MDSCs. More recently, the combination of MDSC targeting with immune checkpoint inhibitor treatment has been applied effectively to several preclinical tumor models and cancer patients. An example of this combinatory approach that is relevant to pulmonary hypertension research is the use of PD-1 blockade with phenformin, an antidiabetic drug from the biguanide class. In one study, phenformin was able to enhance the effect of immune checkpoint inhibition, as evidenced by an increase in CD8 ^+^ T cell infiltration in a melanoma model [184]. Although biguanides have previously been associated with metabolic acidosis-induced pulmonary vasoconstriction [185], more recent evidence has demonstrated protection against pulmonary hypertension development by another drug in the class, metformin, in multiple models of disease [186,187]. Intriguingly, metformin alters tumor bed PMN-MDSC accumulation by facilitating an increase in relevant chemokines—primarily CXCL1—signaling [188].

Finally, in order to elicit immune escape, MDSCs must first be recruited to the tumor or inflammatory site. Therefore, antagonism of CXCR2 has been demonstrated to work in combination with traditional chemotherapeutic agents in decreasing cellular senescence and malignancy, primarily through a decrease in MDSC tumor infiltration [189]. Similarly, blockade of interaction of chemokine receptor CCR5, primarily expressed on Mo-MDSC, with its ligands (CCL3, CCL4, and CCL5) significantly improved survival of melanoma-bearing animals [190]. The use of similar agents in combination with drugs that inhibit MDSC function or frequency could be potentially useful in the treatment of pulmonary hypertension.

## 5. Conclusions

The parallels between the pathogenesis of pulmonary hypertension and cancer are extrapolative but have largely been borne out experimentally in multiple animal models and clinical settings. Although best studied in the context of malignancy, MDSCs represent a novel and exciting area of research in the field of pulmonary vascular disease. The role of MDSCs, and PMN-MDSCs in particular, fits neatly into the vast knowledge base previously established on the role of the immune system in the pathogenesis of pulmonary hypertension. While myeloid cells will likely not be a panacea for pulmonary hypertension [191], the major advantage for continued study is the large amount of readily applied translational drugs targeting MDSC-related pathways for use in patients with disease [192]. 

## Figures and Tables

**Table 1:** Comparison of potential effectors in disease pathogenesis of pulmonary hypertension (PH) and myeloid-derived suppressor cell (MDSC) mediated pathology.

Molecular or Cellular Effector	Pulmonary Hypertension [ref.]	Myeloid-Derived Suppressor Cells (MDSCs) [ref.]
CXCR2/IL–8	**⇧** [63,64]	**⇧** [10,76,77,78,79]
Arg1	**⇧** [9,82,83,84,85]	⇩ [86] and ⇧ [87,88,89]
iNOS	**⇧** [91,92,95]	**⇧** [99]
IDO	**⇩** [97,98,99]	⇧ [100,101,102,103]
STAT3	**⇧** [105,106]	**⇧** [107]
HIF	**⇧** [110,111,112]	⇧ [116,117] (via PD–1/PD–L1 axis)
DC	**⇧** [118,119,120,121,122]	**⇧** [122,123,124,125,126,127] (immature DC)
MΦ	**⇧** [52,57,81,131] (M1 phenotype)	⇧ [135,136] (M2/TAM phenotype)
Treg	⇩ [139,141,144,148] ⇧ [137,138,140]	**⇩** [150,151] **⇧** [104,149]
T_H_17	**⇧** [68,145,146]	⇩ [149] and ⇧ [150,151]

Abbreviations: CXCR2 (C–X–C motif chemokine receptor type 2); IL–8 (interleukin 8); Arg1 (arginase–1); iNOS (inducible nitric oxide synthase); IDO (indoleamine–pyrrole 2,3–dioxygenase); STAT3 (signal transducer and activator of transcription 3); HIF (hypoxia-inducible factor); PD–1 (programmed cell death protein–1); PD–L1 (programmed death–ligand 1); DC (dendritic cell); MΦ (macrophage, either M1 [inflammatory] or M2 [reparative] polarized); TAM (tumor-associated macrophage); Treg (regulatory T cell); T_H_17 (T helper 17 cell).

**Table 2:** Potential therapies targeting myeloid-derived suppressor cell (MDSC) in treatment of pulmonary hypertension (PH).

Drug(s)	Mechanism or Pathway of Action	Expected Outcome
Sildenafil Tadalafil	Phosphodiesterase-5 inhibitor; downregulate Arg1 and iNOS expression in MDSC	In addition to vasodilatory effects, inhibits MDSC–mediated immunosuppression
All–Trans Retinoic Acid (ATRA)	Retinoic acid signal transduction	Differentiation of MDSC into macrophages and DC, and decrease collagen deposition
AZD9150	STAT3 antisense oligonucleotide inhibitor	Inhibition of MDSC immunosuppressive activity and restoration of T cell function
Metformin Phenformin	Antidiabetic drug of biguanide class	Blocks accumulation of MDSC and enhances effect of PD-1 blockade
Nivolumab Pembrolizumab Atezolizumab	Monoclonal antibodies directed against immune checkpoint inhibitors PD–1 or PD–L1	Decreased T cell exhaustion, arrest, and anergy
AZD5059	CXCR2 antagonist	Decreased MDSC trafficking to site of inflammation and injury

Abbreviations: CXCR2 (C–X–C motif chemokine receptor type 2); Arg1 (arginase–1); iNOS (inducible nitric oxide synthase); STAT3 (signal transducer and activator of transcription 3); PD–1 (programmed cell death protein–1); PD–L1 (programmed death-ligand 1); DC (dendritic cell).

## Abbreviations

CXCR2	C-X-C motif chemokine receptor type 2
IL–8	Interleukin 8
PD-1	Programmed cell death protein-1
PD-L1	Programmed death-ligand 1
Arg1	Arginase-1
iNOS	Inducible nitric oxide synthase
IDO	Indoleamine-pyrrole 2,3-dioxygenase
HIF	Hypoxia-inducible factor
STAT3	Signal transducer and activator of transcription 3
PMN-MDSC	Polymorphonuclear myeloid-derived suppressor cell
Mo-MDSC	Monocytic myeloid-derived suppressor cell
DC	Dendritic cell
Mϕ	Macrophage
Treg	Regulatory T cell
T_H_17	T helper 17 cell
VEGF	Vascular endothelial growth factor
IL-10	Interleukin 10
IL-6	Interleukin 6
IL-23	Interleukin 23
TGF-β	Transforming growth factor beta
